# Supplementary material for: Incidence of anogenital warts after the introduction of the quadrivalent HPV vaccine program in Manitoba, Canada
Source: PLoS One. 2022 Apr 26;17(4):e0267646. doi: 10.1371/journal.pone.0267646 (PMC9041799; doi:10.1371/journal.pone.0267646)
Supplement: S1 Table — (PDF) [file pone.0267646.s001.pdf]

S1 Table: Tariff codes used to identify a person with anogenital warts in the Medical Services Database.

Code	Description
3372	Anus, condyloma, single or multiple, internal or external, destruction, in hospital
3433	Anus, condyloma, external, electrodessication, initial, per sitting
3434	Anus, condyloma, external, electrodessication, subsequent, per sitting
4120	Penis, penile skin lesion, including warts, local excision or fulguration, per sitting
4412	Vulva, condylomata excision or destruction any method less than 10 warts up to 25% of vulva
4413	Vulva, condylomata excision or destruction any method 10 or more warts more than 25% of vulva
4415	Vagina, condylomata excision or destruction any method less than 5 warts up to 25% of vagina
4416	Vagina, condylomata excision or destruction any method 5 or more warts more than 25% of vagina
4422	Vulva, condyloma accuminata local excision, fulguration, chemical application or injection or other treatment, per sitting
4427	Vulva, condyloma accuminata, extensive removal under general anaesthesia
4430	Vulva, condylomata excision or destruction any method less than 10 warts up to 25% of vulva
4432	Vulva, condylomata excision or destruction any method 10 or more warts more than 25% of vulva
4472	Vagina, condylomata excision or destruction any method less than 10 warts up to 25% of vagina
4475	Vagina, condylomata excision or destruction any method 10 or more warts more than 25% of vagina
