# Supplementary material for: Incidence of anogenital warts after the introduction of the quadrivalent HPV vaccine program in Manitoba, Canada
Source: PLoS One. 2022 Apr 26;17(4):e0267646. doi: 10.1371/journal.pone.0267646 (PMC9041799; doi:10.1371/journal.pone.0267646)
Supplement: S2 Table — (PDF) [file pone.0267646.s002.pdf]

S2 Table: Identification of a person with anogenital warts from the Hospital Abstracts database.

Date	ICD version	Criteria[1]
04/1994-03/2004	ICD-9-CM	078.11 diagnosis OR (078.10 / 078.19 diagnosis AND related procedure in Supplementary table 3)
>03/2004	ICD-10-CA	A63.0 diagnosis OR (B07 diagnosis AND related procedure in Supplementary table 4)
