# Supplementary material for: Incidence of anogenital warts after the introduction of the quadrivalent HPV vaccine program in Manitoba, Canada
Source: PLoS One. 2022 Apr 26;17(4):e0267646. doi: 10.1371/journal.pone.0267646 (PMC9041799; doi:10.1371/journal.pone.0267646)
Supplement: S3 Table — (PDF) [file pone.0267646.s003.pdf]

S3 Table: ICD-9-CM procedure codes used to assist in the identification of a person with anogenital warts.

Code	Description
48.82	Excision of perirectal tissue
49.04	Other excision of perianal tissue
49.3	Local excision or destruction of other lesion or tissue of anus
58.3	Excision or destruction of lesion or tissue of urethra
61.3	Excision or destruction of lesion or tissue of scrotum
64.2	Local excision or destruction of lesion of penis
67.32	Destruction of lesion of cervix by cauterization – electroconization of cervix
67.33	Destruction of lesion of cervix by cryosurgery – cryoconization of cervix
67.39	Other excision or destruction of lesion or tissue of cervix
70.33	Excision or destruction of lesion of vagina
71.3	Local excision or destruction of vulva and perineum
