# Supplementary material for: Incidence of anogenital warts after the introduction of the quadrivalent HPV vaccine program in Manitoba, Canada
Source: PLoS One. 2022 Apr 26;17(4):e0267646. doi: 10.1371/journal.pone.0267646 (PMC9041799; doi:10.1371/journal.pone.0267646)
Supplement: S4 Table — (PDF) [file pone.0267646.s004.pdf]

S4 Table: ICD-10-CA procedure codes used to assist in the identification of a person with anogenital warts.

Code	Description
1.RS.59.CA.GX	Destruction vagina using per orifice approach and device NEC
1.NT.59.CA.GX	Destruction anus using per orifice approach and device NEC
1.PQ.59.LA.GX	Destruction urethra using open approach and device NEC
1.RW.59.JA.GX	Destruction vulva using external approach and device NEC
1.RW.59.JA.X7	Destruction vulva chemocautery agent
1.RY.87.LA	Excision, partial perineum
