# Supplementary material for: Incidence of anogenital warts after the introduction of the quadrivalent HPV vaccine program in Manitoba, Canada
Source: PLoS One. 2022 Apr 26;17(4):e0267646. doi: 10.1371/journal.pone.0267646 (PMC9041799; doi:10.1371/journal.pone.0267646)
Supplement: S5 Table — (PDF) [file pone.0267646.s005.pdf]

S5 Table: Tariff codes for treatment of anogenital warts used to assist in the identification of extended care for a person with anogenital warts in combination with an ICD-9 code of 078.

**Code**	**Description**
0253	Excision & simple closure – single lesion, any location
0254	Excision & simple closure – each additional lesion to a maximum of four
0255	Excision & closure – multiple lesions, extensive
0397	Laser vaporization, other than face, one lesion
0398	Laser vaporization, other than face, two lesions
0399	Laser vaporization, other than face, three or more lesions
0401	Cautery (electro, chemo, or simple surgical excision, one lesion) elsewhere
0402	Warts & fibrocutanous tags - simple
0404	Cryocautery, etc., of benign lesion of skin, etc., second lesion
0405	Cryocautery, etc., of benign lesion of skin, etc., subsequent lesions (each)
0406	Cryocautery, etc., of benign lesion of skin, etc., complicated lesions
3300	Rectum, villous papilloma of rectum, extensive, local excision
3301	Rectum, unlisted or unusually complicated
3311	Rectum, proctosigmoidoscopy
3315	Rectum, proctosigmoidoscopy with removal of polyp or papilloma, single
3317	Rectum, proctosigmoidoscopy with removal of polyp or papilloma, multiple
3429	Anus, unlisted or unusually complicated
3994	Urethroscopy, therapeutic, polyps, urethral, excision of fulguration with or without urethroscopy
4000	Urethra, urethroscopy, diagnostic, initial or subsequent
4221	Scrotum, skin lesion, local excision
4229	Scrotum, unlisted or unusually complicated
4611	Cervix, local excision of lesion, cauterization of biopsy, one or more sites
4641	Cryosurgery of the cervix for other conditions
8470	General practice visit – regional gynaecological exam – including cytological smear - cervix
8471	General practice visit – regional gynaecological exam – no cytological smear
8495	Obstetrics / gynaecology visit – complete gynaecological exam – including cytological smear - cervix
8496	Obstetrics / gynaecology visit – regional gynaecological exam – including cytological smear - cervix
8497	Obstetrics / gynaecology visit – regional gynaecological exam – no cytological smear
8498	General practice visit – complete gynaecological exam – including cytological smear - cervix
8499	General practice visit – complete gynaecological exam – no cytological smear
8501	Office visits, regional, history and examination
8502	Office visits, complete or extensive re-examination for same illness
8507	Office visits, subsequent visit
8509	Office visits, regional or subsequent visit or well baby care
8529	Office visits, regional intermediate visit or subsequent visit or well baby care
