# Supplementary material for: Incidence of anogenital warts after the introduction of the quadrivalent HPV vaccine program in Manitoba, Canada
Source: PLoS One. 2022 Apr 26;17(4):e0267646. doi: 10.1371/journal.pone.0267646 (PMC9041799; doi:10.1371/journal.pone.0267646)
Supplement: S6 Table — (PDF) [file pone.0267646.s006.pdf]

S6 Table: Age-standardized incidence rate per 100,000 person-years (95% confidence interval) of certain conditions by year and gender.

Year	Anogenital warts		AGW-related prescription		Chlamydia		Gonorrhea	
	Female	Male	Female	Male	Female	Male	Female	Male
2001	119 (110-128)	129 (120-138)	30 (26-35)	55 (49-61)	382 (366-397)	144 (135-154)	55 (49-61)	57 (51-63)
2002	99 (91-107)	122 (113-131)	44 (39-50)	63 (56-69)	387 (372-403)	149 (139-159)	51 (45-57)	50 (44-56)
2003	101 (93-109)	125 (116-134)	64 (57-70)	78 (71-86)	416 (399-432)	167 (157-177)	73 (66-80)	66 (59-72)
2004	111 (103-120)	133 (123-142)	94 (86-102)	122 (113-131)	449 (432-466)	213 (201-224)	87 (79-94)	86 (79-94)
2005	115 (106-123)	140 (130-149)	101 (93-109)	124 (115-134)	384 (369-399)	218 (206-230)	93 (85-101)	94 (87-102)
2006	116 (107-125)	136 (126-145)	103 (95-111)	129 (120-138)	414 (398-430)	242 (230-255)	118 (109-126)	131 (121-140)
2007	111 (102-119)	126 (117-135)	106 (98-114)	109 (100-117)	554 (536-573)	299 (285-312)	125 (116-134)	107 (99-115)
2008	118 (110-127)	138 (128-147)	105 (97-113)	142 (133-152)	704 (684-725)	345 (331-360)	116 (107-124)	93 (85-100)
2009	112 (103-120)	140 (131-150)	139 (130-149)	174 (164-185)	624 (604-643)	316 (302-329)	88 (81-96)	68 (62-75)
2010	104 (96-112)	149 (140-159)	128 (119-137)	154 (144-164)	623 (604-642)	325 (311-339)	83 (76-90)	64 (57-70)
2011	107 (99-115)	147 (137-156)	110 (102-118)	138 (128-147)	648 (629-668)	333 (319-347)	91 (84-99)	66 (59-72)
2012	104 (96-112)	145 (136-154)	104 (96-112)	121 (112-130)	630 (611-648)	318 (304-331)	110 (102-118)	85 (78-92)
2013	102 (94-110)	128 (120-137)	92 (85-99)	101 (94-109)	607 (589-626)	301 (288-314)	100 (93-108)	75 (69-82)
2014	88 (81-95)	137 (128-146)	79 (72-86)	106 (98-113)	586 (568-604)	306 (293-319)	87 (80-95)	69 (63-76)
2015	88 (81-96)	137 (128-146)	63 (57-70)	96 (89-104)	615 (596-633)	310 (297-323)	88 (81-95)	66 (60-73)
2016	86 (79-93)	131 (123-140)	40 (35-44)	65 (59-71)	592 (574-610)	308 (295-321)	165 (155-175)	135 (127-144)
2017	81 (74-88)	127 (118-135)	39 (34-43)	57 (51-62)	428 (413-443)	227 (216-238)	149 (140-158)	129 (120-137)
