# Supplementary material for: Incidence of anogenital warts after the introduction of the quadrivalent HPV vaccine program in Manitoba, Canada
Source: PLoS One. 2022 Apr 26;17(4):e0267646. doi: 10.1371/journal.pone.0267646 (PMC9041799; doi:10.1371/journal.pone.0267646)
Supplement: S7 Table — (PDF) [file pone.0267646.s007.pdf]

S7 Table: Crude incidence rate per 100,000 person-years (95% confidence interval) of certain conditions among 14-18 year-olds by year and gender.

Year	Anogenital warts		AGW-related prescription		Chlamydia		Gonorrhea	
	Female	Male	Female	Male	Female	Male	Female	Male
2001	236 (191-288)	49 (31-75)	70 (46-100)	40 (23-64)	1,872 (1,740-2,010)	444 (383-512)	231 (186-283)	82 (57-114)
2002	170 (133-216)	54 (34-81)	79 (54-112)	54 (34-81)	1,977 (1,843-2,119)	433 (373-500)	227 (183-279)	112 (83-149)
2003	207 (165-256)	72 (49-102)	97 (70-133)	46 (28-72)	2,093 (1,956-2,238)	441 (381-508)	395 (336-460)	146 (112-187)
2004	190 (150-237)	55 (35-82)	120 (89-159)	55 (35-82)	2,156 (2,017-2,302)	530 (464-603)	409 (350-475)	174 (137-218)
2005	206 (165-255)	52 (33-79)	114 (84-151)	59 (39-87)	1,748 (1,624-1,879)	465 (403-533)	415 (356-481)	164 (128-207)
2006	184 (145-230)	68 (46-97)	130 (98-169)	84 (59-116)	1,876 (1,748-2,011)	566 (498-641)	512 (446-585)	227 (185-276)
2007	182 (144-227)	45 (27-69)	133 (101-172)	49 (31-75)	2,431 (2,286-2,584)	656 (583-735)	502 (437-574)	217 (176-265)
2008	180 (142-225)	58 (38-85)	86 (61-119)	69 (47-98)	3,039 (2,876-3,208)	775 (695-861)	483 (420-554)	172 (136-215)
2009	210 (169-258)	69 (47-98)	147 (113-188)	83 (58-114)	2,788 (2,633-2,951)	852 (769-942)	331 (279-391)	118 (89-155)
2010	181 (143-226)	49 (31-75)	127 (95-166)	81 (56-112)	2,810 (2,653-2,974)	772 (693-858)	355 (301-416)	137 (104-175)
2011	192 (152-238)	61 (40-88)	109 (80-145)	67 (45-96)	2,787 (2,630-2,951)	816 (734-904)	341 (288-401)	170 (134-213)
2012	171 (134-215)	95 (68-128)	131 (98-170)	63 (42-91)	2,830 (2,672-2,995)	816 (734-905)	496 (431-568)	156 (121-197)
2013	125 (94-165)	50 (31-76)	80 (55-112)	50 (31-76)	2,582 (2,430-2,742)	776 (695-862)	418 (358-485)	202 (163-249)
2014	95 (68-130)	62 (41-90)	83 (58-116)	57 (37-84)	2,223 (2,081-2,373)	650 (577-730)	332 (279-393)	142 (109-182)
2015	42 (24-67)	37 (21-60)	59 (38-88)	67 (45-96)	2,389 (2,241-2,544)	734 (655-819)	317 (265-377)	127 (96-165)
2016	15 (5-32)	39 (23-63)	29 (15-51)	16 (6-33)	2,280 (2,136-2,432)	625 (553-704)	615 (541-696)	225 (183-274)
2017	29 (15-51)	34 (19-57)	32 (17-54)	27 (14-48)	1,592 (1,472-1,719)	465 (403-533)	408 (348-475)	204 (164-251)
