# Supplementary material for: Incidence of anogenital warts after the introduction of the quadrivalent HPV vaccine program in Manitoba, Canada
Source: PLoS One. 2022 Apr 26;17(4):e0267646. doi: 10.1371/journal.pone.0267646 (PMC9041799; doi:10.1371/journal.pone.0267646)
Supplement: S8 Table — (PDF) [file pone.0267646.s008.pdf]

S8 Table: Crude incidence rate per 100,000 person-years (95% confidence interval) of certain conditions among 19-23 year-olds by year and gender.

Year	Anogenital warts		AGW-related prescription		Chlamydia		Gonorrhea	
	**Female**	**Male**	**Female**	**Male**	**Female**	**Male**	**Female**	**Male**
2001	561 (488-641)	287 (237-345)	81 (55-115)	97 (68-133)	2,300 (2,151-2,457)	813 (727-908)	284 (233-343)	234 (189-287)
2002	480 (414-554)	341 (286-403)	129 (96-170)	125 (93-165)	2,320 (2,171-2,477)	871 (782-968)	253 (205-308)	238 (192-291)
2003	490 (423-564)	395 (336-461)	166 (128-211)	158 (122-202)	2,497 (2,343-2,658)	1,007 (912-1,110)	360 (303-424)	326 (273-387)
2004	543 (473-621)	365 (309-429)	221 (178-273)	206 (164-255)	2,711 (2,552-2,878)	1,295 (1,187-1,410)	450 (387-521)	400 (341-466)
2005	570 (499-649)	473 (409-544)	214 (171-264)	218 (175-268)	2,365 (2,217-2,520)	1,248 (1,143-1,361)	488 (422-561)	354 (299-416)
2006	570 (498-648)	419 (359-487)	218 (175-269)	228 (184-279)	2,516 (2,364-2,676)	1,256 (1,150-1,369)	614 (540-696)	507 (440-580)
2007	549 (480-626)	385 (327-449)	264 (216-318)	216 (174-266)	3,193 (3,022-3,371)	1,686 (1,563-1,815)	686 (608-771)	474 (410-545)
2008	579 (508-657)	441 (380-510)	249 (203-301)	198 (158-245)	3,869 (3,681-4,063)	1,880 (1,751-2,016)	550 (481-626)	453 (391-523)
2009	487 (423-558)	376 (320-438)	235 (192-286)	217 (175-265)	3,484 (3,309-3,666)	1,700 (1,579-1,827)	506 (441-579)	307 (257-364)
2010	428 (369-494)	424 (365-488)	228 (185-277)	192 (153-237)	3,364 (3,194-3,540)	1,719 (1,599-1,844)	367 (312-428)	243 (199-293)
2011	448 (388-515)	434 (376-499)	231 (188-280)	201 (162-246)	3,607 (3,433-3,787)	1,771 (1,651-1,897)	435 (376-501)	283 (237-337)
2012	405 (349-467)	432 (376-495)	186 (149-230)	170 (135-211)	3,470 (3,302-3,644)	1,605 (1,494-1,722)	614 (545-690)	335 (286-391)
2013	387 (332-447)	365 (314-423)	137 (105-175)	140 (109-177)	3,306 (3,144-3,475)	1,504 (1,398-1,616)	493 (432-561)	347 (297-403)
2014	325 (275-380)	418 (363-479)	124 (94-160)	137 (107-174)	3,327 (3,164-3,496)	1,527 (1,421-1,640)	500 (438-568)	337 (288-392)
2015	278 (232-331)	344 (294-399)	80 (56-110)	117 (89-152)	3,453 (3,285-3,626)	1,498 (1,392-1,610)	431 (374-495)	319 (272-373)
2016	270 (225-322)	324 (276-378)	69 (47-98)	81 (58-110)	3,221 (3,060-3,389)	1,529 (1,422-1,642)	789 (710-874)	479 (420-544)
2017	230 (188-278)	258 (215-307)	46 (28-70)	54 (36-79)	2,370 (2,232-2,515)	1,083 (994-1,179)	748 (671-831)	525 (463-592)
