# Supplementary material for: Incidence of anogenital warts after the introduction of the quadrivalent HPV vaccine program in Manitoba, Canada
Source: PLoS One. 2022 Apr 26;17(4):e0267646. doi: 10.1371/journal.pone.0267646 (PMC9041799; doi:10.1371/journal.pone.0267646)
Supplement: S9 Table — (PDF) [file pone.0267646.s009.pdf]

S9 Table: Crude incidence rates (per 100,000 person-years; 95% confidence interval) of AGW-related prescription among birth cohorts by age and gender.

Group / birth year	1993	1994	1995	1996	1997	1998	1999	2000
Female 13 year-olds	60 (20-140)	72 (26-156)	72 (26-156)	149 (77-260)	77 (28-167)	76 (28-166)	64 (21-149)	77 (28-167)
Female 14 year-olds	71 (26-155)	71 (26-155)	95 (41-187)	49 (13-125)	127 (61-233)	126 (60-232)	89 (36-183)	25 (3-91)
Female 15 year-olds	71 (26-155)	70 (26-153)	82 (33-169)	36 (7-106)	37 (8-110)	37 (8-109)	113 (52-214)	63 (20-146)
Female 16 year-olds	129 (65-231)	58 (19-135)	58 (19-135)	71 (26-155)	62 (20-145)	49 (13-126)	99 (43-195)	37 (8-108)
Female 17 year-olds	151 (81-259)	126 (63-226)	193 (113-309)	106 (48-201)	73 (27-159)	61 (20-141)	61 (20-141)	36 (7-106)
Female 18 year-olds	138 (71-240)	167 (94-276)	101 (46-191)	115 (55-212)	12 (0-66)	0 (0-43)	94 (41-185)	N/A
Female 19 year-olds	211 (127-329)	153 (84-257)	142 (76-244)	79 (32-163)	23 (3-83)	23 (3-82)	N/A	N/A
Female 20 year-olds	131 (67-228)	162 (90-267)	129 (67-226)	99 (45-188)	34 (7-98)	N/A	N/A	N/A
Female 21 year-olds	97 (44-183)	43 (12-109)	106 (51-195)	43 (12-111)	N/A	N/A	N/A	N/A
Female 22 year-olds	107 (51-197)	32 (7-92)	63 (23-136)	N/A	N/A	N/A	N/A	N/A
Female 23 year-olds	63 (23-137)	83 (36-164)	N/A	N/A	N/A	N/A	N/A	N/A
Male 13 year-olds	68 (25-148)	80 (32-164)	47 (13-120)	117 (56-214)	12 (0-67)	84 (34-173)	24 (3-86)	24 (3-87)
Male 14 year-olds	33 (7-98)	45 (12-116)	46 (13-118)	92 (40-182)	95 (41-187)	47 (13-121)	71 (26-154)	71 (26-155)
Male 15 year-olds	55 (18-129)	56 (18-130)	34 (7-100)	57 (18-133)	70 (26-153)	35 (7-103)	23 (3-84)	35 (7-103)
Male 16 year-olds	66 (24-143)	77 (31-159)	22 (3-81)	56 (18-131)	23 (3-84)	58 (19-135)	46 (13-118)	0 (0-43)
Male 17 year-olds	32 (7-95)	98 (45-186)	55 (18-128)	77 (31-159)	11 (0-64)	57 (19-133)	23 (3-82)	11 (0-63)
Male 18 year-olds	86 (37-169)	64 (23-139)	54 (17-126)	97 (44-184)	89 (38-175)	33 (7-97)	22 (3-78)	N/A
Male 19 year-olds	52 (17-121)	72 (29-149)	84 (36-165)	95 (43-180)	32 (7-94)	53 (17-123)	N/A	N/A
Male 20 year-olds	142 (77-237)	111 (55-199)	81 (35-161)	71 (29-147)	41 (11-105)	N/A	N/A	N/A
Male 21 year-olds	119 (62-208)	159 (91-258)	110 (55-197)	70 (28-144)	N/A	N/A	N/A	N/A
Male 22 year-olds	109 (54-194)	88 (40-168)	50 (16-116)	N/A	N/A	N/A	N/A	N/A
Male 23 year-olds	68 (27-140)	68 (27-141)	N/A	N/A	N/A	N/A	N/A	N/A
