# Supplementary material for: Incidence of anogenital warts after the introduction of the quadrivalent HPV vaccine program in Manitoba, Canada
Source: PLoS One. 2022 Apr 26;17(4):e0267646. doi: 10.1371/journal.pone.0267646 (PMC9041799; doi:10.1371/journal.pone.0267646)
Supplement: S10 Table — (PDF) [file pone.0267646.s010.pdf]

S10 Table: Crude incidence rates (per 100,000 person-years; 95% confidence interval) of chlamydia among birth cohorts by age and gender.

Group / birth year	1993	1994	1995	1996	1997	1998	1999	2000
Female 13 year-olds	144 (75-252)	72 (26-156)	72 (26-156)	124 (59-228)	128 (61-236)	140 (70-251)	64 (21-149)	204 (117-332)
Female 14 year-olds	440 (310-606)	285 (182-423)	487 (349-660)	379 (258-538)	368 (246-528)	290 (184-435)	570 (416-763)	377 (255-539)
Female 15 year-olds	1,148 (931-1,400)	1,349 (1,114-1,619)	1,268 (1,040-1,531)	1,425 (1,179-1,706)	1,237 (1,005-1,505)	1,271 (1,036-1,543)	753 (574-969)	1,301 (1,063-1,576)
Female 16 year-olds	2,338 (2,024-2,686)	2,415 (2,098-2,767)	2,239 (1,934-2,578)	2,687 (2,348-3,061)	1,983 (1,687-2,315)	1,884 (1,598-2,208)	2,066 (1,764-2,404)	2,016 (1,719-2,349)
Female 17 year-olds	3,539 (3,152-3,960)	3,154 (2,793-3,550)	3,183 (2,821-3,578)	3,197 (2,828-3,600)	2,728 (2,382-3,110)	2,922 (2,565-3,315)	2,531 (2,200-2,899)	1,687 (1,419-1,991)
Female 18 year-olds	4,504 (4,069-4,972)	3,889 (3,492-4,319)	3,513 (3,136-3,924)	3,341 (2,968-3,749)	3,073 (2,709-3,471)	3,388 (3,009-3,802)	1,947 (1,662-2,267)	N/A
Female 19 year-olds	4,556 (4,126-5,019)	4,296 (3,882-4,743)	3,617 (3,237-4,029)	4,202 (3,786-4,652)	3,727 (3,332-4,155)	2,868 (2,525-3,243)	N/A	N/A
Female 20 year-olds	3,817 (3,428-4,238)	4,061 (3,662-4,493)	3,639 (3,262-4,049)	3,743 (3,356-4,163)	2,787 (2,452-3,156)	N/A	N/A	N/A
Female 21 year-olds	3,510 (3,140-3,912)	3,550 (3,179-3,953)	3,406 (3,044-3,799)	2,314 (2,013-2,646)	N/A	N/A	N/A	N/A
Female 22 year-olds	3,113 (2,765-3,492)	3,031 (2,691-3,402)	2,246 (1,956-2,567)	N/A	N/A	N/A	N/A	N/A
Female 23 year-olds	2,719 (2,398-3,072)	2,433 (2,131-2,766)	N/A	N/A	N/A	N/A	N/A	N/A
Male 13 year-olds	0 (0-42)	46 (12-117)	0 (0-43)	12 (0-65)	0 (0-44)	0 (0-44)	12 (0-66)	0 (0-44)
Male 14 year-olds	11 (0-62)	68 (25-148)	69 (25-151)	35 (7-101)	48 (13-122)	59 (19-138)	82 (33-170)	24 (3-86)
Male 15 year-olds	211 (127-329)	301 (198-438)	251 (157-380)	159 (87-268)	270 (171-405)	223 (134-348)	81 (33-168)	164 (90-275)
Male 16 year-olds	417 (295-572)	496 (362-664)	506 (369-677)	753 (584-956)	616 (462-806)	394 (273-551)	553 (408-733)	323 (215-467)
Male 17 year-olds	747 (582-946)	981 (789-1,206)	901 (717-1,119)	795 (622-1,002)	856 (673-1,073)	810 (632-1,021)	757 (587-961)	339 (229-485)
Male 18 year-olds	1,508 (1,269-1,778)	1,275 (1,057-1,525)	1,270 (1,052-1,521)	1,100 (897-1,336)	1,220 (1,003-1,470)	994 (799-1,221)	780 (611-983)	N/A
Male 19 year-olds	1,356 (1,134-1,609)	1,491 (1,258-1,756)	1,326 (1,105-1,577)	1,506 (1,270-1,774)	1,409 (1,179-1,670)	1,065 (867-1,294)	N/A	N/A

Male 20 year-olds	1,698 (1,451-1,975)	1,443 (1,216-1,699)	1,487 (1,255-1,748)	1,670 (1,424-1,946)	1,137 (935-1,369)	N/A	N/A	N/A
Male 21 year-olds	1,691 (1,446-1,965)	1,571 (1,335-1,836)	1,492 (1,262-1,752)	1,072 (878-1,295)	N/A	N/A	N/A	N/A
Male 22 year-olds	1,391 (1,171-1,640)	1,583 (1,348-1,847)	1,221 (1,015-1,457)	N/A	N/A	N/A	N/A	N/A
Male 23 year-olds	1,273 (1,064-1,510)	1,043 (855-1,260)	N/A	N/A	N/A	N/A	N/A	N/A
