# Supplementary material for: Incidence of anogenital warts after the introduction of the quadrivalent HPV vaccine program in Manitoba, Canada
Source: PLoS One. 2022 Apr 26;17(4):e0267646. doi: 10.1371/journal.pone.0267646 (PMC9041799; doi:10.1371/journal.pone.0267646)
Supplement: S11 Table — (PDF) [file pone.0267646.s011.pdf]

S11 Table: Crude incidence rates (per 100,000 person-years; 95% confidence interval) of gonorrhea among birth cohorts by age and gender.

Group / birth year	1993	1994	1995	1996	1997	1998	1999	2000
Female 13 year-olds	48 (13-123)	36 (7-105)	12 (0-67)	25 (3-90)	13 (0-71)	0 (0-47)	13 (0-71)	64 (21-149)
Female 14 year-olds	95 (41-187)	24 (3-86)	59 (19-138)	98 (42-193)	25 (3-92)	63 (20-147)	127 (61-233)	101 (43-198)
Female 15 year-olds	189 (108-307)	281 (180-419)	164 (90-276)	254 (157-388)	200 (114-325)	237 (143-370)	125 (60-231)	125 (60-230)
Female 16 year-olds	329 (219-475)	290 (188-429)	313 (206-456)	547 (400-729)	248 (151-383)	382 (259-542)	346 (230-501)	565 (414-754)
Female 17 year-olds	524 (382-701)	333 (223-478)	648 (491-839)	552 (406-735)	365 (247-522)	436 (306-604)	763 (586-976)	374 (254-530)
Female 18 year-olds	470 (337-637)	624 (471-810)	593 (444-776)	438 (310-601)	333 (222-482)	821 (640-1,037)	493 (355-666)	N/A
Female 19 year-olds	721 (556-918)	623 (472-807)	570 (426-747)	508 (371-680)	989 (791-1,222)	759 (589-964)	N/A	N/A
Female 20 year-olds	740 (574-938)	474 (344-636)	474 (344-636)	782 (610-986)	795 (621-1,002)	N/A	N/A	N/A
Female 21 year-olds	623 (473-805)	480 (350-642)	804 (633-1,006)	782 (612-985)	N/A	N/A	N/A	N/A
Female 22 year-olds	417 (297-570)	874 (696-1,083)	700 (542-889)	N/A	N/A	N/A	N/A	N/A
Female 23 year-olds	651 (499-835)	738 (577-931)	N/A	N/A	N/A	N/A	N/A	N/A
Male 13 year-olds	0 (0-42)	11 (0-63)	12 (0-65)	0 (0-43)	0 (0-44)	0 (0-44)	12 (0-66)	0 (0-44)
Male 14 year-olds	0 (0-41)	34 (7-99)	23 (3-83)	12 (0-64)	24 (3-86)	47 (13-121)	47 (13-120)	0 (0-44)
Male 15 year-olds	44 (12-113)	45 (12-114)	34 (7-100)	57 (18-133)	59 (19-137)	59 (19-137)	12 (0-65)	35 (7-103)
Male 16 year-olds	110 (53-202)	88 (38-174)	124 (62-221)	45 (12-115)	198 (115-316)	104 (48-198)	115 (55-212)	127 (63-227)
Male 17 year-olds	130 (67-227)	207 (125-324)	176 (101-286)	276 (179-408)	183 (104-297)	114 (55-210)	271 (174-404)	170 (95-280)
Male 18 year-olds	289 (190-420)	298 (198-430)	258 (166-384)	227 (140-346)	255 (162-383)	408 (288-563)	390 (273-540)	N/A
Male 19 year-olds	259 (167-382)	280 (184-407)	313 (211-447)	200 (121-313)	438 (314-594)	348 (239-489)	N/A	N/A

Male 20 year-olds	334 (230-468)	333 (229-468)	438 (317-590)	316 (214-448)	430 (310-581)	N/A	N/A	N/A
Male 21 year-olds	388 (276-530)	288 (193-414)	611 (467-785)	511 (380-672)	N/A	N/A	N/A	N/A
Male 22 year-olds	276 (184-399)	472 (348-626)	606 (463-778)	N/A	N/A	N/A	N/A	N/A
Male 23 year-olds	486 (361-640)	643 (497-818)	N/A	N/A	N/A	N/A	N/A	N/A
