# Supplementary material for: Incidence of anogenital warts after the introduction of the quadrivalent HPV vaccine program in Manitoba, Canada
Source: PLoS One. 2022 Apr 26;17(4):e0267646. doi: 10.1371/journal.pone.0267646 (PMC9041799; doi:10.1371/journal.pone.0267646)
Supplement: S12 Table — (PDF) [file pone.0267646.s012.pdf]

S12 Table: Incidence rate ratios (95% confidence interval) of certain conditions for cohorts 1 year before and after the introduction of school-based qHPV vaccination (birth cohort 1997 vs 1996) by gender.

**Condition**	**16 year-olds**	**17 year-olds**	**18 year-olds**	**16-18 year-olds**
Anogenital warts				
Female	0.35 (0.09-1.28)	0.35 (0.13-0.95)	0.32 (0.12-0.88)	0.34 (0.18-0.63)
Male	0.52 (0.09-2.82)	1.24 (0.38-4.06)	0.41 (0.13-1.31)	0.65 (0.32-1.34)
AGW-related prescription				
Female	0.87 (0.27-2.85)	0.69 (0.25-1.94)	0.10 (0.01-0.81)	0.50 (0.25-0.99)
Male	0.41 (0.08-2.13)	0.15 (0.02-1.20)	0.91 (0.35-2.37)	0.54 (0.26-1.12)
Chlamydia				
Female	0.74 (0.60-0.90)	0.85 (0.72-1.02)	0.92 (0.78-1.09)	0.85 (0.76-0.94)
Male	0.82 (0.57-1.17)	1.08 (0.78-1.49)	1.11 (0.85-1.45)	1.02 (0.85-1.22)
Gonorrhea				
Female	0.45 (0.27-0.77)	0.66 (0.42-1.05)	0.76 (0.47-1.24)	0.62 (0.47-0.82)
Male	4.40 (1.48-13.06)	0.66 (0.35-1.24)	1.13 (0.62-2.03)	1.16 (0.79-1.69)
