# Supplementary material for: Incidence of anogenital warts after the introduction of the quadrivalent HPV vaccine program in Manitoba, Canada
Source: PLoS One. 2022 Apr 26;17(4):e0267646. doi: 10.1371/journal.pone.0267646 (PMC9041799; doi:10.1371/journal.pone.0267646)
Supplement: S13 Table — (PDF) [file pone.0267646.s013.pdf]

S13 Table: Incidence rate ratios (95% confidence interval) of certain conditions for cohorts 3 years before and after the introduction of school-based qHPV vaccination (birth cohorts 1997-1999 vs 1994-1996) by gender.

**Condition**	**16 year-olds**	**17 year-olds**	**18 year-olds**	**16-18 year-olds**
Anogenital warts				
Female	0.31 (0.13-0.72)	0.20 (0.10-0.41)	0.21 (0.11-0.40)	0.23 (0.15-0.34)
Male	0.31 (0.09-1.13)	0.76 (0.38-1.52)	0.47 (0.27-0.84)	0.53 (0.35-0.80)
AGW-related prescription				
Female	1.12 (0.57-2.22)	0.46 (0.25-0.82)	0.28 (0.13-0.58)	0.51 (0.35-0.73)
Male	0.81 (0.37-1.79)	0.39 (0.17-0.89)	0.67 (0.33-1.34)	0.60 (0.39-0.93)
Chlamydia				
Female	0.81 (0.72-0.91)	0.86 (0.78-0.95)	0.78 (0.71-0.86)	0.82 (0.77-0.87)
Male	0.89 (0.71-1.12)	0.90 (0.75-1.09)	0.82 (0.70-0.96)	0.86 (0.78-0.96)
Gonorrhea				
Female	0.85 (0.63-1.15)	1.02 (0.80-1.30)	1.00 (0.79-1.25)	0.97 (0.84-1.12)
Male	1.62 (0.96-2.74)	0.86 (0.59-1.26)	1.35 (0.99-1.83)	1.20 (0.97-1.49)
