# Supplementary material for: Incidence of anogenital warts after the introduction of the quadrivalent HPV vaccine program in Manitoba, Canada
Source: PLoS One. 2022 Apr 26;17(4):e0267646. doi: 10.1371/journal.pone.0267646 (PMC9041799; doi:10.1371/journal.pone.0267646)
Supplement: S14 Table — (PDF) [file pone.0267646.s014.pdf]

S14 Table: Crude incidence rate per 100,000 person-years (95% confidence interval) of certain conditions among 15-19 year-olds by year and gender.

Year	Anogenital warts		AGW-related prescription		Chlamydia		Gonorrhea	
	Female	Male	Female	Male	Female	Male	Female	Male
2001	355 (299-418)	78 (54-110)	77 (53-110)	45 (27-70)	2,266 (2,121-2,418)	611 (538-690)	275 (226-331)	125 (94-164)
2002	263 (215-318)	89 (63-123)	94 (67-129)	68 (46-98)	2,455 (2,304-2,612)	638 (564-718)	283 (233-340)	158 (122-200)
2003	300 (249-358)	131 (99-170)	130 (98-170)	61 (40-89)	2,612 (2,458-2,774)	660 (585-741)	460 (396-530)	222 (180-272)
2004	306 (255-364)	102 (74-137)	177 (139-223)	72 (49-102)	2,782 (2,623-2,948)	815 (732-905)	527 (459-602)	242 (197-293)
2005	331 (278-391)	110 (81-146)	154 (118-196)	78 (54-109)	2,219 (2,078-2,367)	729 (651-813)	482 (418-554)	251 (206-302)
2006	279 (231-335)	114 (85-150)	142 (108-183)	109 (81-145)	2,336 (2,193-2,487)	795 (714-883)	611 (538-690)	319 (268-376)
2007	269 (222-323)	95 (68-128)	161 (126-204)	72 (49-102)	3,068 (2,904-3,239)	997 (906-1,094)	625 (552-705)	303 (254-359)
2008	293 (244-349)	101 (73-135)	123 (92-161)	92 (66-124)	3,886 (3,702-4,077)	1,118 (1,022-1,220)	588 (518-666)	264 (218-316)
2009	289 (241-344)	108 (80-143)	192 (153-238)	126 (95-163)	3,486 (3,313-3,667)	1,136 (1,040-1,239)	407 (349-472)	159 (125-200)
2010	263 (217-316)	97 (71-130)	159 (124-202)	88 (63-120)	3,446 (3,273-3,625)	1,093 (998-1,193)	423 (364-489)	181 (144-225)
2011	283 (235-338)	104 (76-138)	153 (118-195)	104 (76-138)	3,553 (3,377-3,736)	1,139 (1,043-1,242)	448 (387-516)	228 (186-276)
2012	238 (195-289)	158 (124-199)	153 (118-194)	79 (55-109)	3,529 (3,354-3,711)	1,090 (997-1,191)	595 (524-672)	208 (169-255)
2013	220 (178-269)	84 (60-116)	108 (79-144)	64 (43-92)	3,294 (3,124-3,470)	1,059 (967-1,159)	539 (471-613)	248 (204-299)
2014	171 (134-215)	114 (85-150)	107 (78-143)	65 (43-93)	2,919 (2,758-3,087)	912 (826-1,005)	442 (381-510)	212 (171-259)
2015	84 (59-117)	79 (55-109)	72 (49-103)	67 (45-96)	3,067 (2,900-3,240)	1,004 (913-1,102)	387 (330-452)	195 (157-241)
2016	41 (24-65)	74 (51-104)	34 (18-56)	25 (12-44)	2,896 (2,735-3,064)	931 (843-1,025)	744 (663-831)	306 (257-363)
2017	53 (33-80)	56 (36-83)	38 (22-62)	31 (17-53)	2,057 (1,922-2,199)	678 (604-759)	577 (507-655)	273 (227-326)
