# Supplementary material for: Incidence of anogenital warts after the introduction of the quadrivalent HPV vaccine program in Manitoba, Canada
Source: PLoS One. 2022 Apr 26;17(4):e0267646. doi: 10.1371/journal.pone.0267646 (PMC9041799; doi:10.1371/journal.pone.0267646)
Supplement: S15 Table — (PDF) [file pone.0267646.s015.pdf]

S15 Table: Crude incidence rate per 100,000 person-years (95% confidence interval) of certain conditions among 20-24 year-olds by year and gender.

Year	Anogenital warts		AGW-related prescription		Chlamydia		Gonorrhea	
	Female	Male	Female	Male	Female	Male	Female	Male
2001	521 (451-599)	349 (293-414)	76 (51-110)	119 (87-159)	1,985 (1,846-2,132)	751 (667-842)	266 (217-323)	233 (187-286)
2002	443 (379-515)	393 (334-461)	133 (99-175)	148 (113-192)	2,000 (1,861-2,146)	787 (701-880)	209 (165-260)	222 (178-274)
2003	435 (372-506)	421 (359-490)	142 (107-184)	184 (144-231)	2,119 (1,976-2,269)	925 (833-1,025)	314 (261-375)	282 (232-340)
2004	500 (433-576)	445 (382-515)	176 (137-223)	221 (178-272)	2,313 (2,165-2,469)	1,166 (1,063-1,277)	386 (326-452)	383 (325-449)
2005	521 (452-597)	549 (479-626)	189 (148-237)	240 (195-293)	2,096 (1,956-2,244)	1,140 (1,038-1,249)	466 (401-538)	307 (255-366)
2006	530 (461-606)	466 (402-538)	207 (165-257)	248 (202-301)	2,217 (2,073-2,368)	1,205 (1,101-1,317)	555 (484-633)	486 (421-559)
2007	521 (454-596)	412 (352-479)	253 (207-307)	239 (194-291)	2,848 (2,686-3,017)	1,585 (1,465-1,711)	630 (555-712)	463 (400-534)
2008	540 (471-616)	505 (439-579)	235 (190-287)	215 (173-265)	3,357 (3,181-3,539)	1,780 (1,653-1,913)	496 (430-569)	423 (363-491)
2009	497 (432-570)	442 (381-510)	209 (168-258)	231 (187-281)	3,032 (2,868-3,204)	1,669 (1,548-1,796)	476 (412-547)	306 (256-363)
2010	413 (355-478)	486 (423-555)	224 (181-273)	219 (178-267)	3,046 (2,884-3,215)	1,586 (1,471-1,707)	339 (286-398)	239 (196-290)
2011	426 (367-491)	501 (438-571)	193 (154-238)	199 (160-245)	3,165 (3,002-3,334)	1,683 (1,566-1,806)	379 (324-440)	247 (204-297)
2012	409 (353-471)	467 (408-532)	169 (133-211)	179 (143-221)	3,092 (2,934-3,257)	1,574 (1,464-1,690)	554 (488-626)	354 (303-412)
2013	348 (297-406)	421 (366-482)	125 (95-162)	156 (123-195)	2,849 (2,699-3,006)	1,440 (1,336-1,549)	420 (364-483)	352 (302-409)
2014	302 (254-355)	472 (413-536)	103 (76-137)	156 (123-195)	2,960 (2,808-3,119)	1,539 (1,432-1,652)	424 (367-487)	318 (270-371)
2015	296 (249-349)	395 (342-454)	70 (48-98)	136 (105-172)	3,113 (2,956-3,276)	1,507 (1,402-1,618)	414 (358-476)	277 (233-327)
2016	271 (226-322)	379 (327-437)	64 (43-91)	99 (74-131)	2,974 (2,820-3,134)	1,509 (1,404-1,620)	730 (655-812)	518 (457-585)
2017	241 (198-289)	287 (242-338)	49 (31-73)	57 (38-83)	2,170 (2,039-2,308)	1,060 (972-1,154)	660 (589-738)	531 (469-599)
