# Supplementary material for: Incidence of anogenital warts after the introduction of the quadrivalent HPV vaccine program in Manitoba, Canada
Source: PLoS One. 2022 Apr 26;17(4):e0267646. doi: 10.1371/journal.pone.0267646 (PMC9041799; doi:10.1371/journal.pone.0267646)
Supplement: S16 Table — (PDF) [file pone.0267646.s016.pdf]

S16 Table: Crude incidence rate per 100,000 person-years (95% confidence interval) of certain conditions among 25-29 year-olds by year and gender.

Year	Anogenital warts		AGW-related prescription		Chlamydia		Gonorrhea	
	Female	Male	Female	Male	Female	Male	Female	Male
2001	281 (230-341)	414 (352-485)	70 (46-102)	163 (125-209)	820 (731-918)	449 (384-522)	155 (118-201)	203 (160-254)
2002	226 (180-280)	367 (308-434)	67 (44-99)	110 (79-149)	804 (715-900)	464 (397-539)	156 (118-202)	137 (102-180)
2003	237 (190-292)	332 (276-396)	72 (47-105)	145 (109-189)	839 (748-936)	453 (387-526)	176 (136-224)	137 (102-180)
2004	259 (210-315)	405 (343-475)	132 (98-174)	161 (123-207)	932 (838-1,035)	604 (527-688)	188 (146-237)	258 (209-314)
2005	253 (205-309)	423 (359-494)	133 (99-176)	171 (132-219)	748 (663-841)	711 (629-802)	200 (157-250)	270 (220-328)
2006	306 (253-367)	385 (325-453)	169 (130-216)	181 (140-229)	947 (852-1,051)	727 (644-819)	280 (229-338)	390 (330-459)
2007	276 (226-334)	372 (314-438)	165 (127-211)	182 (142-230)	1,306 (1,194-1,425)	908 (815-1,009)	289 (238-348)	271 (221-328)
2008	304 (252-363)	461 (396-533)	142 (107-184)	257 (209-312)	1,722 (1,595-1,857)	1,089 (989-1,198)	324 (270-385)	247 (200-301)
2009	272 (223-328)	462 (398-533)	146 (111-188)	227 (183-279)	1,320 (1,210-1,437)	904 (814-1,002)	188 (148-235)	190 (150-238)
2010	303 (253-361)	526 (459-601)	138 (105-179)	222 (179-272)	1,506 (1,391-1,628)	1,048 (951-1,151)	231 (188-282)	159 (123-203)
2011	260 (214-313)	417 (358-483)	115 (85-152)	213 (172-262)	1,471 (1,358-1,591)	1,073 (977-1,176)	246 (201-297)	201 (161-249)
2012	247 (203-298)	451 (390-518)	125 (94-162)	224 (182-273)	1,446 (1,336-1,562)	984 (893-1,081)	279 (232-333)	270 (223-323)
2013	284 (237-338)	357 (304-417)	82 (58-113)	141 (109-180)	1,504 (1,393-1,622)	979 (890-1,075)	248 (205-299)	203 (164-249)
2014	184 (147-228)	387 (332-448)	65 (44-93)	166 (131-208)	1,435 (1,327-1,548)	1,000 (911-1,095)	215 (175-262)	216 (176-263)
2015	221 (180-267)	390 (336-450)	83 (59-113)	131 (100-167)	1,600 (1,488-1,718)	956 (870-1,048)	250 (207-300)	211 (171-256)
2016	218 (178-263)	370 (318-428)	43 (27-66)	97 (71-129)	1,617 (1,506-1,734)	942 (858-1,032)	507 (446-575)	428 (372-490)
2017	227 (187-273)	337 (287-392)	37 (22-58)	105 (79-138)	1,132 (1,040-1,230)	763 (688-844)	507 (446-574)	416 (361-477)
