# Supplementary material for: Incidence of anogenital warts after the introduction of the quadrivalent HPV vaccine program in Manitoba, Canada
Source: PLoS One. 2022 Apr 26;17(4):e0267646. doi: 10.1371/journal.pone.0267646 (PMC9041799; doi:10.1371/journal.pone.0267646)
Supplement: S17 Table — (PDF) [file pone.0267646.s017.pdf]

S17 Table: Crude incidence rate per 100,000 person-years (95% confidence interval) of certain conditions among 30-39 year-olds by year and gender.

Year	Anogenital warts		AGW-related prescription		Chlamydia		Gonorrhea	
	Female	Male	Female	Male	Female	Male	Female	Male
2001	143 (119-171)	251 (218-288)	31 (20-46)	100 (79-124)	212 (182-246)	129 (105-156)	34 (22-49)	87 (68-110)
2002	140 (116-169)	240 (208-277)	32 (21-47)	89 (70-112)	186 (157-218)	123 (100-149)	50 (36-68)	69 (52-90)
2003	122 (99-149)	233 (201-270)	63 (47-83)	90 (70-113)	232 (199-268)	160 (134-191)	45 (32-63)	100 (79-124)
2004	131 (106-158)	254 (220-292)	79 (61-102)	143 (117-172)	241 (207-278)	218 (187-254)	64 (47-84)	116 (93-142)
2005	128 (104-156)	232 (199-269)	80 (62-103)	119 (96-146)	245 (211-282)	259 (225-298)	73 (55-94)	151 (125-181)
2006	143 (118-173)	274 (238-314)	86 (67-109)	146 (120-175)	261 (226-300)	319 (280-362)	100 (79-125)	203 (173-238)
2007	116 (93-143)	268 (233-307)	76 (58-98)	116 (93-143)	387 (344-433)	346 (306-390)	108 (86-134)	141 (115-170)
2008	141 (116-170)	221 (189-257)	55 (40-74)	142 (117-171)	586 (534-643)	406 (362-453)	117 (94-143)	149 (123-178)
2009	146 (120-175)	263 (228-301)	89 (69-112)	131 (107-159)	492 (444-543)	345 (305-388)	82 (64-105)	108 (87-134)
2010	122 (99-149)	269 (234-308)	90 (70-113)	159 (132-189)	461 (415-510)	410 (367-457)	85 (66-107)	101 (80-125)
2011	144 (119-173)	278 (243-317)	89 (70-112)	130 (106-157)	536 (487-589)	407 (364-453)	105 (84-130)	87 (67-109)
2012	144 (119-172)	266 (232-304)	68 (52-89)	107 (86-132)	502 (456-553)	398 (356-444)	77 (59-98)	119 (97-145)
2013	142 (118-169)	227 (196-262)	71 (55-92)	99 (79-123)	501 (455-551)	383 (343-427)	99 (79-123)	92 (73-115)
2014	144 (120-172)	234 (203-268)	65 (49-84)	106 (86-131)	541 (493-592)	418 (376-463)	78 (60-99)	88 (69-110)
2015	130 (107-156)	254 (222-290)	63 (47-81)	120 (98-145)	507 (462-556)	448 (405-495)	100 (80-123)	103 (83-127)
2016	144 (120-170)	240 (209-274)	46 (33-62)	80 (63-101)	499 (455-547)	416 (375-460)	183 (156-213)	228 (198-261)
2017	128 (106-153)	240 (209-274)	41 (29-57)	54 (40-71)	405 (365-448)	353 (316-393)	186 (160-216)	229 (199-262)
