# Supplementary material for: Incidence of anogenital warts after the introduction of the quadrivalent HPV vaccine program in Manitoba, Canada
Source: PLoS One. 2022 Apr 26;17(4):e0267646. doi: 10.1371/journal.pone.0267646 (PMC9041799; doi:10.1371/journal.pone.0267646)
Supplement: Attachment — Submitted filename: PLOSone-reply.pdf [file pone.0267646.s018.pdf]

**Editorial and Reviewer comments**

We thank the Editor and Reviewers for their careful review of our manuscript and for the constructive remarks and advice. We know that these reviews are done by busy scientists on pro bono basis, and we truly appreciate their valuable service.

**<u>Replies to Editorial comments</u>**

1: Please ensure that your manuscript meets PLOS ONE's style requirements, including those for file naming.
**A1: We followed the instructions in the two linked guides.**

2: Please ensure that you have specified (1) whether consent was informed, (2) what type you obtained (for instance, written or verbal, and if verbal, how it was documented and witnessed). If your study included minors, state whether you obtained consent from parents or guardians. If the need for consent was waived by the ethics committee and (3) If you are reporting a retrospective study of medical records or archived samples, please ensure that you have discussed whether all data were fully anonymized before you accessed them and/or whether the IRB or ethics committee waived the requirement for informed consent. If patients provided informed written consent to have data from their medical records used in research, please include this information.
**A2: We added the following to the end of the methods: "The REB waived the requirement for informed consent in this retrospective study of medical records, because the data did not contain any direct identifiers."**

3-5: We note that the grant information you provided in the 'Funding Information' and 'Financial Disclosure' sections do not match. When you resubmit, please ensure that you provide the correct grant numbers for the awards you received for your study in the 'Funding Information' section

Please provide an amended statement that declares *all* the funding or sources of support (whether external or internal to your organization) received during this study, as detailed online in our guide for authors at http://journals.plos.org/plosone/s/submit-now. Please also include the statement "There was no additional external funding received for this study." in your updated Funding Statement. Please include your amended Funding Statement within your cover letter. We will change the online submission form on your behalf.

We note that you have provided funding information that is not currently declared in your Funding Statement. However, funding information should not appear in the Acknowledgments section or other areas of your manuscript. We will only publish funding information present in the Funding Statement section of the online submission form.

Please remove any funding-related text from the manuscript and let us know how you would like to update your Funding Statement. Please include your amended statements within your cover letter; we will change the online submission form on your behalf.
**A3-5: We removed the section from the manuscript, the corrected statement, incorporating your comments above is:**
**This work was supported by the Merck Investigator Studies Program (IIS #51109) with a grant to the International Centre for Infectious Diseases (ICID). The sponsor had no role in the design or conduct of the study, including but not limited to, data identification, collection, management, analysis and**

**interpretation, or preparation, review, or approval of the results. The opinions presented in the report do not necessarily reflect those of the sponsor. SMM's work is supported, in part, by funding from the Canada Research Chair Program (#231458). There was no additional external funding received for this study.**

6: Competing interest. Please confirm that this does not alter your adherence to all PLOS ONE policies on sharing data and materials, by including the following statement: ""This does not alter our adherence to PLOS ONE policies on sharing data and materials." (as detailed online in our guide for authors http://journals.plos.org/plosone/s/competing-interests). If there are restrictions on sharing of data and/or materials, please state these. Please note that we cannot proceed with consideration of your article until this information has been declared.
**A6: The corrected competing interest section (which we have removed from the manuscript should be:**

**CHR has received an unrestricted research grant from Pfizer for an unrelated study. KW does not have a financial relationship to disclose. EK has received consulting fees from Merck Canada and GlaxoSmithKline for unrelated studies. EK has received honoraria and travel expenses from Merck Canada. SMM received research funding from Assurex, GSK, Merck, Pfizer, Roche and Sanofi for unrelated studies and is/was a member of advisory boards for GSK, Merck, Sanofi and Seqirus. This does not alter our adherence to PLOS ONE policies on sharing data and materials.**

7-8: In your Data Availability statement, you have not specified where the minimal data set underlying the results described in your manuscript can be found. PLOS defines a study's minimal data set as the underlying data used to reach the conclusions drawn in the manuscript and any additional data required to replicate the reported study findings in their entirety. All PLOS journals require that the minimal data set be made fully available. For more information about our data policy, please see http://journals.plos.org/plosone/s/data-availability.

Upon re-submitting your revised manuscript, please upload your study's minimal underlying data set as either Supporting Information files or to a stable, public repository and include the relevant URLs, DOIs, or accession numbers within your revised cover letter. For a list of acceptable repositories, please see http://journals.plos.org/plosone/s/data-availability#loc-recommended-repositories. Any potentially identifying patient information must be fully anonymized.

Important: If there are ethical or legal restrictions to sharing your data publicly, please explain these restrictions in detail. Please see our guidelines for more information on what we consider unacceptable restrictions to publicly sharing data: http://journals.plos.org/plosone/s/data-availability#loc-unacceptable-data-access-restrictions. Note that it is not acceptable for the authors to be the sole named individuals responsible for ensuring data access.

We will update your Data Availability statement to reflect the information you provide in your cover letter.

We note that you have indicated that data from this study are available upon request. PLOS only allows data to be available upon request if there are legal or ethical restrictions on sharing data publicly. For more information on unacceptable data access restrictions, please see http://journals.plos.org/plosone/s/data-availability#loc-unacceptable-data-access-restrictions.

In your revised cover letter, please address the following prompts:

a) If there are ethical or legal restrictions on sharing a de-identified data set, please explain them in detail (e.g., data contain potentially sensitive information, data are owned by a third-party organization, etc.) and who has imposed them (e.g., an ethics committee). Please also provide contact information for a data access committee, ethics committee, or other institutional body to which data requests may be sent.

b) If there are no restrictions, please upload the minimal anonymized data set necessary to replicate your study findings as either Supporting Information files or to a stable, public repository and provide us with the relevant URLs, DOIs, or accession numbers. For a list of acceptable repositories, please see http://journals.plos.org/plosone/s/data-availability#loc-recommended-repositories.

We will update your Data Availability statement on your behalf to reflect the information you provide.
**A7-8: The corrected Data Availability statement (which we removed from the manuscript) is:**

**Data used in this article was derived from administrative health and social data as a secondary use. The data was provided under specific data sharing agreements only for approved use at Manitoba Centre for Health Policy (MCHP). The original source data is not owned by the researchers or MCHP and as such cannot be provided to a public repository. The original data source and approval for use has been noted in the acknowledgments of the article. Where necessary, source data specific to this article or project may be reviewed at MCHP with the consent of the original data providers, along with the required privacy and ethical review bodies. Because this data consists of personal health data of residents of Manitoba, its disclosure is governed by The Personal Health Information Act (PHIA), which legally bans the disclosure of the source data or derived data to a public repository. Anyone wishing to access this information should contact Manitoba Health's Health Information Privacy Committee (see https://www.gov.mb.ca/health/hipc/index.html for contact info).**

<u>**Replies to Reviewer 2**</u>

Q: Since now we also have data on the impact of vaccination on the incidence of cervical cancer, this data may be included in the Discussion of the manuscript.
**A: We have included this. Thank you for the suggestion.**
